# Bis{2-[6-(1*H*-benzimidazol-2-yl-κ*N* ^3^)-2-pyridyl-κ*N*]benzimidazolato-κ*N*}manganese(II)

**DOI:** 10.1107/S1600536809008411

**Published:** 2009-03-14

**Authors:** Xian-Qun Bai, Shu-Hua Zhang

**Affiliations:** a Key Laboratory of Non-ferrous Metal Materials and Processing Technology, Department of Materials and Chemistry Engineering, Guilin University of Technology, Ministry of Education, Guilin 541004, People’s Republic of China; b College of Pharmacy, Guilin Medical University, Guilin 541004, People’s Republic of China

## Abstract

In the title compound, [Mn(C_19_H_12_N_5_)_2_], each Mn^II^ atom lies on a position of site symmetry 222 and has a distorted octa­hedral coordination geometry made up from six N atoms of two tridentate 2-[6-(1*H*-benzimidazol-2-yl)-2-pyrid­yl]benz­imidazolate ligands. The complex mol­ecules are linked into layers parallel to (001) by N—H⋯N hydrogen bonds, with the H atoms disordered over four symmetry-equivalent non-coordinated N atoms.

## Related literature

For a previous report of this complex, see: Shi *et al.* (2003 ▶). For other comparable transition-metal complexes, see: Harvey *et al.* (2003 ▶); Wang *et al.* (1994 ▶); Yue *et al.* (2006 ▶); Zhang *et al.* (2007 ▶).
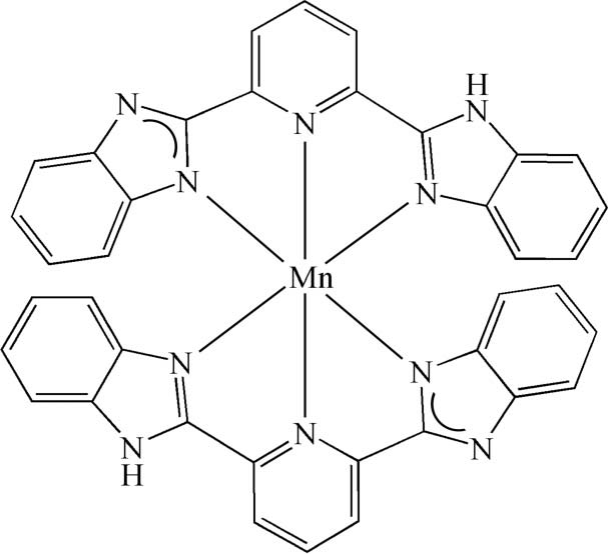


## Experimental

### 

#### Crystal data

[Mn(C_19_H_12_N_5_)_2_]
*M* *_r_* = 675.61
Tetragonal, 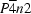

*a* = 10.1225 (14) Å
*c* = 15.865 (3) Å
*V* = 1625.6 (5) Å^3^
*Z* = 2
Mo *K*α radiation
μ = 0.45 mm^−1^
*T* = 298 K
0.45 × 0.44 × 0.31 mm

#### Data collection

Bruker SMART 2K CCD diffractometer
Absorption correction: none
6803 measured reflections
1599 independent reflections
1226 reflections with *I* > 2σ(*I*)
*R*_int_ = 0.040

#### Refinement

*R*[*F* ^2^ > 2σ(*F* ^2^)] = 0.035
*wR*(*F* ^2^) = 0.095
*S* = 1.04
1599 reflections
113 parameters
H-atom parameters constrained
Δρ_max_ = 0.18 e Å^−3^
Δρ_min_ = −0.21 e Å^−3^
Absolute structure: Flack (1983 ▶), 701 Friedel pairs
Flack parameter: 0.00 (1)

### 

Data collection: *SMART* (Bruker, 2003 ▶); cell refinement: *SAINT* (Bruker, 2003 ▶); data reduction: *SAINT*; program(s) used to solve structure: *SHELXS97* (Sheldrick, 2008 ▶); program(s) used to refine structure: *SHELXL97* (Sheldrick, 2008 ▶); molecular graphics: *SHELXTL* (Sheldrick, 2008 ▶); software used to prepare material for publication: *SHELXTL*.

## Supplementary Material

Crystal structure: contains datablocks I, global. DOI: 10.1107/S1600536809008411/bi2351sup1.cif

Structure factors: contains datablocks I. DOI: 10.1107/S1600536809008411/bi2351Isup2.hkl

Additional supplementary materials:  crystallographic information; 3D view; checkCIF report

## Figures and Tables

**Table 1:** Hydrogen-bond geometry (Å, °)

*D*—H⋯*A*	*D*—H	H⋯*A*	*D*⋯*A*	*D*—H⋯*A*
N3—H1⋯N3^i^	0.91	1.89	2.736 (4)	154

Symmetry code: (i) 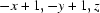.

**Fig. 1.**
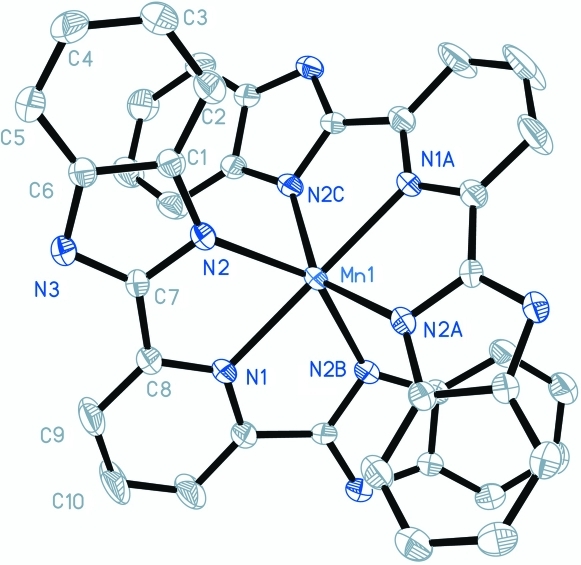
Molecular structure showing 30% probability displacement ellipsoids. H atoms are omitted. Symmetry codes: (A) -x + 2, -y, z; (B) -y + 1, -x + 1, -z; (C) y + 1, x - 1, -z.

## supplementary crystallographic information

### Comment

The 2,6-bis(1*H*-benzimidazol-2-yl)pyridine ligand is known to form complexes with various transition metal atoms (Harvey *et al.*, 2003; Wang *et al.*, 1994; Yue *et al.*, 2006; Zhang *et al.*, 2007). The title compound, containing Mn^II^, has been reported previously (Shi *et al.*, 2003), with closely comparable cell parameters but refined in space group *Pn*. Atomic coordinates were not reported and they are not available in the Cambridge Structural Database. However, diagrams of the structure in the paper of Shi *et al.* (2003) suggest it to be closely comparable to the current reported structure, and it is probable that the previous refinement in *Pn* is an instance of "missed symmetry".

### Experimental

Manganese nitrate hexahydrate (0.144 g, 0.5 mmol) and 2,6-bis(1*H*-benzimidazol-2-yl)pyridine (0.1536 g, 1 mmol) were dissolved in ethanol (8 ml). The solution was placed in a 15 ml Teflon-lined stainless steel bomb and heated at 423 K for 96 h. The cooled mixture yielded red block-shaped crystals in about 41% yield. The crystals were washed with ethanol and dried in air.

### Refinement

All H atoms were positioned geometrically (C—H = 0.93 Å, N—H = 0.91 Å) and refined as riding with *U*_iso_(H) = 1.2 *U*_eq_(C or N). The site occupancy of H1 was constrained to 0.5 so that it sums to a total of 2 H atoms disordered over the four symmetry-equivalent N3 atoms.

### Crystal data

**Table:**

[Mn(C_19_H_12_N_5_)_2_]	*D*_x_ = 1.380 Mg m^−^^3^
*M**_r_* = 675.61	Mo *K*α radiation, λ = 0.71073 Å
Tetragonal, *P*4*n*2	Cell parameters from 6803 reflections
Hall symbol: P -4 -2n	θ = 2.4–26.0°
*a* = 10.1225 (14) Å	µ = 0.45 mm^−^^1^
*c* = 15.865 (3) Å	*T* = 298 K
*V* = 1625.6 (5) Å^3^	Block, red
*Z* = 2	0.45 × 0.44 × 0.31 mm
*F*(000) = 694	

### Data collection

**Table:**

Bruker SMART 2K CCD diffractometer	1226 reflections with *I* > 2<s(*I*)
Radiation source: fine-focus sealed tube	*R*_int_ = 0.040
graphite	θ_max_ = 26.0°, θ_min_ = 2.4°
φ and ω scans	*h* = −10→12
6803 measured reflections	*k* = −12→11
1599 independent reflections	*l* = −19→12

### Refinement

**Table:**

Refinement on *F*^2^	Secondary atom site location: difference Fourier map
Least-squares matrix: full	Hydrogen site location: inferred from neighbouring sites
*R*[*F*^2^ > 2σ(*F*^2^)] = 0.035	H-atom parameters constrained
*wR*(*F*^2^) = 0.095	*w* = 1/[σ^2^(*F*_o_^2^) + (0.0482*P*)^2^ + 0.3361*P*] where *P* = (*F*_o_^2^ + 2*F*_c_^2^)/3
*S* = 1.03	(Δ/σ)_max_ < 0.001
1599 reflections	Δρ_max_ = 0.18 e Å^−^^3^
113 parameters	Δρ_min_ = −0.21 e Å^−^^3^
0 restraints	Absolute structure: Flack (1983), 701 Friedel pairs
Primary atom site location: structure-invariant direct methods	Flack parameter: 0.00 (1)

### Special details

**Table:**

Geometry. All e.s.d.'s (except the e.s.d. in the dihedral angle between two l.s. planes) are estimated using the full covariance matrix. The cell e.s.d.'s are taken into account individually in the estimation of e.s.d.'s in distances, angles and torsion angles; correlations between e.s.d.'s in cell parameters are only used when they are defined by crystal symmetry. An approximate (isotropic) treatment of cell e.s.d.'s is used for estimating e.s.d.'s involving l.s. planes.
Refinement. Refinement of *F*^2^ against ALL reflections. The weighted *R*-factor *wR* and goodness of fit *S* are based on *F*^2^, conventional *R*-factors *R* are based on *F*, with *F* set to zero for negative *F*^2^. The threshold expression of *F*^2^ > σ(*F*^2^) is used only for calculating *R*-factors(gt) *etc*. and is not relevant to the choice of reflections for refinement. *R*-factors based on *F*^2^ are statistically about twice as large as those based on *F*, and *R*- factors based on ALL data will be even larger.

### Fractional atomic coordinates and isotropic or equivalent isotropic displacement parameters (Å^2^)

**Table:**

	*x*	*y*	*z*	*U*_iso_*/*U*_eq_	Occ. (<1)
Mn1	0.5000	0.0000	0.2500	0.0372 (2)	
C1	0.3191 (3)	0.1985 (3)	0.12293 (17)	0.0379 (6)	
C2	0.2023 (3)	0.1312 (3)	0.1090 (2)	0.0542 (9)	
H2	0.1913	0.0448	0.1279	0.065*	
C3	0.1031 (4)	0.1952 (4)	0.0668 (3)	0.0626 (10)	
H3	0.0234	0.1520	0.0575	0.075*	
C4	0.1195 (3)	0.3226 (4)	0.0376 (2)	0.0625 (10)	
H4	0.0511	0.3624	0.0078	0.075*	
C5	0.2336 (3)	0.3919 (3)	0.0513 (2)	0.0540 (9)	
H5	0.2432	0.4782	0.0321	0.065*	
C6	0.3348 (3)	0.3286 (3)	0.09494 (19)	0.0406 (7)	
C7	0.5083 (3)	0.2688 (3)	0.16267 (18)	0.0366 (7)	
C8	0.6350 (3)	0.2666 (3)	0.2067 (2)	0.0471 (8)	
C9	0.7292 (4)	0.3650 (4)	0.2072 (3)	0.0915 (16)	
H9	0.7147	0.4441	0.1789	0.110*	
C10	0.8439 (3)	0.3439 (3)	0.2500	0.128 (3)	
H10	0.9089	0.4089	0.2500	0.154*	
N1	0.65540 (18)	0.15540 (18)	0.2500	0.0381 (7)	
N2	0.4325 (2)	0.1611 (2)	0.16468 (16)	0.0388 (6)	
N3	0.4560 (2)	0.3722 (2)	0.12179 (17)	0.0437 (6)	
H1	0.5082	0.4448	0.1157	0.066*	0.50

### Atomic displacement parameters (Å^2^)

**Table:**

	*U*^11^	*U*^22^	*U*^33^	*U*^12^	*U*^13^	*U*^23^
Mn1	0.0277 (3)	0.0277 (3)	0.0560 (5)	−0.0078 (3)	0.000	0.000
C1	0.0366 (16)	0.0338 (16)	0.0435 (15)	−0.0021 (11)	−0.0031 (14)	−0.0071 (14)
C2	0.0514 (19)	0.0429 (18)	0.068 (2)	−0.0116 (14)	−0.0116 (18)	−0.0014 (17)
C3	0.049 (2)	0.056 (2)	0.083 (3)	−0.0112 (17)	−0.016 (2)	−0.003 (2)
C4	0.0494 (19)	0.062 (2)	0.076 (3)	0.0047 (18)	−0.0231 (18)	0.0033 (19)
C5	0.053 (2)	0.0375 (17)	0.071 (2)	0.0038 (15)	−0.0036 (17)	0.0058 (18)
C6	0.0381 (16)	0.0316 (16)	0.0520 (18)	−0.0011 (12)	−0.0007 (14)	−0.0043 (14)
C7	0.0358 (15)	0.0240 (13)	0.0501 (18)	−0.0011 (11)	0.0004 (15)	0.0004 (12)
C8	0.0367 (16)	0.0352 (16)	0.069 (2)	−0.0057 (12)	−0.0053 (16)	0.0089 (15)
C9	0.066 (2)	0.051 (2)	0.158 (4)	−0.0324 (18)	−0.046 (3)	0.047 (2)
C10	0.078 (3)	0.078 (3)	0.229 (8)	−0.058 (3)	−0.083 (5)	0.083 (5)
N1	0.0292 (9)	0.0292 (9)	0.0559 (19)	−0.0071 (11)	0.0007 (15)	−0.0007 (15)
N2	0.0347 (13)	0.0285 (11)	0.0531 (15)	−0.0063 (10)	−0.0047 (11)	−0.0010 (11)
N3	0.0402 (13)	0.0285 (12)	0.0623 (16)	−0.0045 (10)	−0.0046 (13)	0.0033 (13)

### Geometric parameters (Å, °)

**Table:**

Mn1—N1^i^	2.225 (3)	C5—C6	1.393 (4)
Mn1—N1	2.225 (3)	C5—H5	0.930
Mn1—N2^ii^	2.226 (2)	C6—N3	1.372 (4)
Mn1—N2^i^	2.226 (2)	C7—N2	1.333 (3)
Mn1—N2	2.226 (2)	C7—N3	1.341 (4)
Mn1—N2^iii^	2.226 (2)	C7—C8	1.461 (4)
C1—N2	1.378 (3)	C8—N1	1.334 (3)
C1—C2	1.382 (4)	C8—C9	1.379 (4)
C1—C6	1.399 (4)	C9—C10	1.362 (4)
C2—C3	1.370 (5)	C9—H9	0.930
C2—H2	0.930	C10—C9^iii^	1.362 (4)
C3—C4	1.380 (5)	C10—H10	0.930
C3—H3	0.930	N1—C8^iii^	1.334 (3)
C4—C5	1.369 (5)	N3—H1	0.910
C4—H4	0.930		
			
N1^i^—Mn1—N1	180.00 (13)	C4—C5—C6	117.7 (3)
N1^i^—Mn1—N2^ii^	72.49 (5)	C4—C5—H5	121.2
N1—Mn1—N2^ii^	107.51 (5)	C6—C5—H5	121.2
N1^i^—Mn1—N2^i^	72.49 (5)	N3—C6—C5	131.7 (3)
N1—Mn1—N2^i^	107.51 (5)	N3—C6—C1	107.8 (3)
N2^ii^—Mn1—N2^i^	144.99 (11)	C5—C6—C1	120.5 (3)
N1^i^—Mn1—N2	107.51 (5)	N2—C7—N3	115.0 (2)
N1—Mn1—N2	72.49 (5)	N2—C7—C8	118.8 (2)
N2^ii^—Mn1—N2	85.43 (12)	N3—C7—C8	126.1 (2)
N2^i^—Mn1—N2	105.11 (12)	N1—C8—C9	120.0 (3)
N1^i^—Mn1—N2^iii^	107.51 (5)	N1—C8—C7	113.2 (2)
N1—Mn1—N2^iii^	72.49 (5)	C9—C8—C7	126.8 (3)
N2^ii^—Mn1—N2^iii^	105.11 (12)	C10—C9—C8	118.6 (4)
N2^i^—Mn1—N2^iii^	85.43 (12)	C10—C9—H9	120.7
N2—Mn1—N2^iii^	144.99 (11)	C8—C9—H9	120.7
N2—C1—C2	130.8 (3)	C9—C10—C9^iii^	121.0 (4)
N2—C1—C6	108.5 (2)	C9—C10—H10	119.5
C2—C1—C6	120.7 (3)	C9^iii^—C10—H10	119.5
C3—C2—C1	118.1 (3)	C8—N1—C8^iii^	121.7 (3)
C3—C2—H2	120.9	C8—N1—Mn1	119.15 (16)
C1—C2—H2	120.9	C8^iii^—N1—Mn1	119.15 (16)
C2—C3—C4	121.2 (3)	C7—N2—C1	104.0 (2)
C2—C3—H3	119.4	C7—N2—Mn1	115.90 (18)
C4—C3—H3	119.4	C1—N2—Mn1	138.51 (18)
C5—C4—C3	121.8 (3)	C7—N3—C6	104.6 (2)
C5—C4—H4	119.1	C7—N3—H1	116.9
C3—C4—H4	119.1	C6—N3—H1	138.2

Symmetry codes: (i) −*x*+1, −*y*, *z*; (ii) −*y*+1/2, −*x*+1/2, −*z*+1/2; (iii) *y*+1/2, *x*−1/2, −*z*+1/2.

### Hydrogen-bond geometry (Å, °)

**Table:**

*D*—H···*A*	*D*—H	H···*A*	*D*···*A*	*D*—H···*A*
N3—H1···N3^iv^	0.91	1.89	2.736 (4)	154

Symmetry codes: (iv) −*x*+1, −*y*+1, *z*.
